# Prevalence and associations of hepatitis C viremia in hemodialysis patients at a tertiary care hospital

**DOI:** 10.4103/0971-4065.53324

**Published:** 2009-04

**Authors:** S. Jasuja, A. K. Gupta, R. Choudhry, V. Kher, D. K. Aggarwal, A. Mishra, M. Agarwal, A. Sarin, M. K. Mishra, V. Raina

**Affiliations:** Department of Nephrology, Indraprastha Apollo Hospital, Delhi, India; 1 Department of Immunology and Molecular Biology, Indraprastha Apollo Hospital, Delhi, India; 2 AHERF, Fortis Hospitals, Delhi, India; 3 Department of Nephrology, Fortis Hospitals, Delhi, India

**Keywords:** Duration of dialysis, hemodialysis, hepatitis C, polymerase chain reaction

## Abstract

Hepatitis C virus (HCV) infection in hemodialysis (HD) is a significant problem. We evaluated the prevalence and associations of HCV viremia in our HD patients. All patients undergoing maintenance HD at our center were tested for HCV RNA by PCR after written informed consent. Detailed history regarding age, sex, and duration of dialysis, frequency of dialysis, blood transfusions in one year, number of dialysis centers, dialyzer reuse/fresh use, and recent laboratory data was recorded. A total of 119 patients (77 males and 42 females) were tested for HCV RNA. Thirty three (27.7%) tested positive. Duration of dialysis was significantly longer in HCV RNA positive group (*P* = 0.001). 45.2% of patients with duration of dialysis more than 16 months were HCV RNA positive while only 7.4% of patients with dialysis duration ≤16 months were HCV RNA positive (*P* < 0.001). In univariate analysis, in HCV RNA group patients, ALT, AST, and GGTP were significantly elevated and albumin was significantly lower. 39% of patients who had dialysis at more than one center were HCV RNA positive as compared to 20% for patients undergoing dialysis at single center (*P* = 0.024). Binary logistic regression analysis showed albumin, duration of dialysis, and serum ALT to be significant variables. Sensitivity and specificity of anti-HCV ELISA was 72.7 and 97.7%, respectively. Prevalence of HCV RNA in the HD population is 27.7%. Duration of dialysis, getting dialysis at more than one center, elevated transaminase levels, and low serum albumin are important associations for HCV RNA positivity.

## Introduction

Liver disease caused by hepatitis C virus (HCV) causes significant morbidity and mortality among patients with endstage renal disease (ESRD) treated with hemodialysis (HD). Prevalence of anti-HCV antibody among HD patients is consistently higher than in general population indicating increased risk of acquiring HCV infection among HD patients. The reported incidence varies from country to country and depends upon type of assay used and execution trends for HD. Currently, third-generation anti-HCV ELISA is largely in use and has shown greater sensitivity and specificity in patients receiving HD.[1] Using third-generation ELISA, prevalence of anti-HCV antibodies among dialysis patients was found to be 42% in France,[2] 75% in Moldavia,[3] and 49% in Syria.[4]

There is wide variation in the prevalence of HCV infection among different dialysis units and countries as shown by Dialysis Outcomes and Practice Patterns Study (DOPPS). Mean HCV facility prevalence was 13.5% and varied among countries from 2.6–22.9%.[5] Incidence and prevalence of HCV infection among patients on dialysis is declining in Western countries. In US, incidence has decreased from 1.7% in 1982 to 0.2% in 1997.[6] This decline is attributed to reduction in post-transfusion HCV infection with nucleic acid testing (NAT) based screening and implementation of universal precautions for infection control. A number of risk factors have been identified for HCV infection among dialysis patients, which include number of blood transfusions, duration of endstage kidney disease, mode of dialysis, and the concurrent prevalence of HCV infection in the dialysis unit. With this background and knowledge, we studied prevalence of HCV infection in our HD patients by HCV RNA polymerase chain reaction (PCR). Simultaneously anti-HCV ELISA was also done for all patients. Association of HCV RNA positivity with various variables was studied.

## Materials and Methods

All patients undergoing maintenance HD at our center for over three-month duration were enrolled in the study. Institutional ethical committee approved the study protocol. Patients with acute renal failure undergoing dialysis, holiday dialysis, and those receiving anti HCV treatment were excluded from the study. Patients were enrolled after written informed consent. They were also explained and counseled regarding implications of positive test. Detailed history regarding age, sex, basic disease leading to chronic kidney disease (CKD), duration of CKD/ESRD, duration of HD, frequency of dialysis, dialysis shifts, blood transfusions in last one year, number of dialysis centers visited, reuse/nonreuse of dialyzer, parenteral iron therapy, jaundice, and hepatitis B vaccination was taken and recorded in a proforma. Data regarding hematological parameters, liver function test, kidney function test, anti-HCV, and HBsAg were recorded from patients' dialysis records. At our center, we do not use dedicated machines for HCV-positive patients and we apply universal precautions for infection control. We do not have a fixed dialyzer reuse policy, and the decision depends upon the ability of the patient to pay for a new dialyzer every time. Dialyzer is reused 4–5 times on an average before discard. Single predialysis blood sample in plain vials was taken for HCV RNA. Roche Amplicor HCV RNA 2.0 performed PCR for HCV RNA (Qualitative). For anti-HCV antibodies, third-generation ELISA was used. All samples were stored in deep freezer at −20 degrees and were tested together in a period of five days.

### Statistical methods

Unpaired student's ‘*t*’ test was applied to compare quantitative parameters between HCV RNA negative and HCV RNA positive group of patients. Pearson's chi-square test and Fisher's exact test were used for qualitative parameters. Binary logistic regression analysis was done taking HCV RNA as a dependant variable and diagnosis, albumin, number of centers, duration of dialysis, and alanine aminotransferase (ALT) as independent variables. Receiver operating characteristics (ROC) curve was drawn to determine the threshold value of duration of dialysis for HCV RNA positivity. SPSS 10.0 version was used for analysis.

## Results

Baseline characteristics of total number of patients (*N* = 119) studied is shown in Table 1. Table 2 shows comparative analysis by student's ‘*t*’ test of two groups, HCV RNA negative and HCV RNA positive. Thirty three patients out of 119 tested positive for HCV RNA. Duration of CKD since its first detection, as well as duration of maintenance HD since its initiation were significantly longer in HCV RNA positive patients (*P* = 0.02 and 0.001, respectively). There was no statistically significant difference between two groups in terms of number of blood transfusions received in previous one year. Values of hemoglobin, hematocrit, and serum bilirubin were not statistically different between two groups. Similarly, values of predialysis urea, creatinine, sodium, potassium, calcium, phosphorus, uric acid, and cholesterol were not statistically different between the two groups.

**Table 1:** Baseline characteristics of both groups

Variable	No.	Percentage
Total no. of cases (*N*)	119	100
Sex		
Males	77	65.0
Females	42	35.0
Diagnosis		
Diabetic	50	43
Nondiabetic	69	57
Frequency of dialysis		
Once a week	5	4.2
Twice a week	81	68.1
Thrice a week	33	27.7
Blood transfusions	65	54.6
Hepatitis B vaccination		
Not done	20	16.8
Done once	89	74.8
Done twice or more	10	8.4
Dialyzer		
Reuse	70	58.8
Nonreuse	49	41.2
Dialyzed at		
One center (ours)	70	58.8
Two centers	42	35.3
Three or more	7	5.9

**Table 2:** Comparison of groups 1 (HCV RNA negative) and 2 (HCV RNA positive)

Parameter	*n*	HCV RNA negative (Mean ± SD)	*n*	HCV RNA positive (Mean ± SD)	*P* value
**Quantitative**					
Age (years)	86	55.9 ± 15.7	33	53.18 ± 15.1	0.387
Duration of detection of CRF (months)	84	35.64 ± 46.4	32	58.78 ± 51.7	0.022
Duration of dialysis (months)	84	19.13 ± 18.6	32	37.6 ± 41.4	0.001
Number of blood transfusion in last one year	86	3.91 ± 8.34	33	4.09 ± 7.3	0.911
Hemoglobin (g/dL)	82	10.41 ± 1.95	32	9.86 ± 2.21	0.199
Hematocrit (%)	39	30.19 ± 6.58	23	30.59 ± 6.80	0.810
ALT IU/L	69	22.8 ± 27.63	29	70.1 ± 91.9	0.011
AST IU/L	67	29.16 ± 62.38	29	79.49 ± 138.34	0.069
GGTP (IU/L)	60	69.95 ± 141.07	27	165.71 ± 193.39	0.026
Albumin (gm/dl)	74	3.90 ± 0.54	30	3.66 ± 0.62	0.049
**Qualitative**		**No.(%)**		**No.(%)**	
Males	86	57 (66.3)	33	20 (60.6)	0.562
Diabetics	86	41 (47.7)	33	9 (27.7)	0.044
Dialyzer reuse	86	48 (55.8)	33	22 (66.7)	0.282
Center >1	86	20 (34.9)	33	19 (57.6)	0.024
ALT IU/L >40	69	6 (8.7)	29	15 (51.7)	<0.001
AST IU/L >40	67	3 (4.5)	29	14 (48.3)	<0.001
Duration of dialysis >16 months	84	34 (40.5)	32	28 (87.5)	<0.001
Anti-HCV antibody positive	86	2 (2.3)	33	24 (72.7)	<0.001

Alanine aminotransferase levels were significantly higher in HCV RNA positive group as compared to HCV RNA negative group (70.1 ± 91.9 vs. 22.8 ± 27.6 IU/L respectively, *P* = 0.01). Similarly, aspartate aminotransferase (AST) was also higher in HCV RNA positive group, however *P*-value did not reach significance level (*P* = 0.07). Gamma glutamyl transpeptidase (GGTP) was also statistically significantly higher in HCV RNA positive group (*P* = 0.02). The heavily right skewed values of AST, ALT, and GGTP were also highly statistically significant with log base 10 transformation (*P* = 0.001, <0.001, and <0.001, respectively). Seventy one point four percent (*n* = 15) of HCV RNA positive patients had ALT > 40 IU/L whereas 81.8% (*n* = 63) of HCV RNA negative patients had ALT < 40 IU/L. One international unit per liter increase in ALT increased odds 1.033 times to have HCV RNA positivity. Sensitivity and specificity for ALT (>40 IU/L) was 51.7 and 91.3%, respectively. Eighty two point four percent (*n* = 14) of HCV RNA positive had AST > 40 IU/L whereas 81.0% (*n* = 64) of HCV RNA negative patients had AST < 40 IU/L. Sensitivity and specificity for AST (>40 IU/L) was 48.3 and 95.5%, respectively.

**Figure 1:**
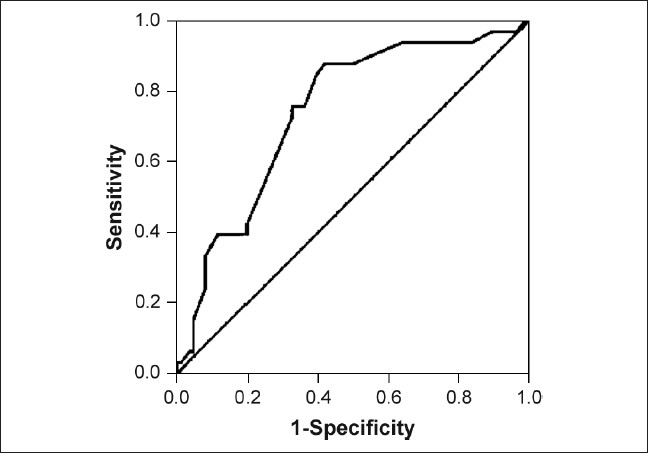
ROC showing cut-off value of 16 months for the variable duration of dialysis

Albumin was significantly lower in HCV RNA positive group as compared to HCV RNA negative group (3.66 ± 0.62 vs. 3.90 ± 0.54 gm/dl, *P* = 0.049). No significant difference between two groups with regard to sex of the patient was observed. The proportion of HCV RNA positivity in diabetics was 18.0% and in nondiabetics it was 34.4% (*P* = 0.044). No statistically significant effect of blood transfusions and hepatitis B vaccination on HCV RNA positivity was observed. There was no impact of dialyzer reuse or nonreuse on HCV RNA positivity. Use of temporary or permanent dialysis access also did not have any impact on HCV RNA positivity. HCV RNA positivity was 20% in group with dialysis at one center (ours), whereas group that had dialysis at more than one center had 39% HCV RNA positivity (*P* = 0.024). History of parenteral iron therapy also did not have an association with HCV RNA positivity. Duration of HD was found to have significant impact on HCV RNA positivity. Only 4 out of 54 patients (7.4%) with duration of dialysis ≤16 months were HCV RNA positive, while 28 out of 62 patients (45.2%) with duration of dialysis > 16 months were HCV RNA positive (*P* < 0.001). The cut-off value of 16 months was calculated from ROC curve [Figure 1]. The distribution of HCV RNA prevalence was also studied according to duration of dialysis. HCV RNA prevalence was highest in patients on dialysis for ≥37 months. One month increase in duration of dialysis, increased odds 1.06 times to have HCV RNA positivity. Odds ratio doubled with one year of dialysis.

Logistic regression analysis was done taking HCV RNA as a dependant variable and others as independent risk factors (diagnosis, albumin, number of centers, duration of dialysis, and ALT) [Table 3]. Analysis was considered only for variables that were significant individually with HCV RNA. For this analysis, 87 out of 119 patients in whom all values of these five independent variables were available were considered. Basic disease diagnosis (diabetic or nondiabetic) and number of centers (one or more than one) were found to be statistically insignificant. Albumin was found to be significant and was negatively associated with HCV RNA positivity. Duration of dialysis and ALT were also significant. Anti-HCV antibody test results were available for all patients (*N* = 119). Twenty six patients were anti-HCV positive of which 24 were HCV RNA positive and two were HCV RNA negative. Nine patients were HCV RNA positive but anti-HCV antibody negative. Sensitivity and specificity of anti-HCV antibodies was found to be 72.7 and 97.7%, respectively.

**Table 3:** Logistic regression analysis

Variable	*n*	Beta coefficient	*P* value	Adjusted odds ratio	95% CI of odd ratio
Diagnosis					
Nondiabetics	52	−1.199	0.l75	0.302	0.053–1.707
Diabetes	35
Albumin	87	−2.516	0.003	0.081	0.015–0.422
No. of centers One	51	−0.158	0.854	0.854	0.217–3.364
More than one	36
Duration of dialysis	87	0.059	0.001	1.06	1.023–1.099
**ALT					
0–40	68	2.795	0	16.356	3.693–72.44
>40	19				

*Considering HCV RNA as dependant variable and others as independent risk factors. (Only variables those were significant individually with HCV RNA);

** Taking cut-off 0–40 as reference (odds equal to 1); patient having more than 40 has increase in the odds to 16.356 and is also significant

## Discussion

Third-generation anti-HCV ELISA is the screening test for the diagnosis of HCV infection. It has shown better performance than the previous two generations of anti-HCV tests with a mean window period of 70 days.[1,7] Detection of HCV RNA by reverse transcriptase PCR has been used as the ‘gold standard’ to identity current HCV infection.[8] PCR can detect HCV RNA within one to three weeks of exposure and prior to the appearance of anti-HCV antibodies or elevation in ALT levels.[9] The qualitative PCR assays are considered most sensitive tests for the diagnosis of HCV infection. We performed HCV RNA PCR (qualitative) as well as anti-HCV ELISA for all patients. Sensitivity and specificity of anti-HCV antibody was found to be 72.7 and 97.7%, respectively. Weinstein *et al.*, reported 94% sensitivity and 91% specificity of third-generation microparticle enzyme immuno assay in identifying HCV RNA positivity.[10]

In general, the greater the elevation in serum ALT, higher is the probability of histological evidence of the liver disease in HCV infection. Among HD patients, serum ALT levels are elevated in 4–67% patients with positive anti-HCV antibodies, 12–31% of patients with positive HCV RNA and one-third of patients with biopsy proven hepatitis.[11] Herrine *et al.*, suggested a lower cut-off value of 18 IU/L for AST and 16 IU/L for ALT which increased sensitivity (61.1%) and specificity (66.7%) for the detection of HCV infection in HD patients.[12] Saab *et al.*, did a prospective study to determine sensitivity, specificity, and predictive values of an elevated ALT level for the diagnosis of HCV infection in HD patients.[13] They reported that a newly elevated ALT is more sensitive and specific for acute HCV infection but its positive predictive value is inadequate. However, a newly elevated ALT level was neither sensitive nor positively predictive of chronic infection. Hence, an elevated ALT level may not be an effective method for screening for HCV infection in HD patients. Our study showed significant correlation of HCV RNA with elevated ALT, AST, and GGTP, and low serum albumin in univariate analysis. In multivariate analysis serum albumin and ALT were again found to be significant risk factors for HCV RNA positivity. One international unit per liter increase in ALT increased odds 1.033 times for HCV RNA positivity.

High prevalence of HCV infection in HD units is a significant risk factor for acquiring HCV infection. The incidence was directly related to the prevalence in the dialysis unit. Units with a prevalence of <19% had an annual incidence of 2.5% compared to a 35.3% incidence in units with a prevalence >60%.[14] Molecular biology techniques have provided evidence of nosocominal transmission of HCV within individual HD units. A study conducted at Belgium HD units used genotyping by sequence analysis of the HCV core region and it revealed that 20 out of 23 seroconverts were infected with same HCV strain.[15] Several reports have suggested cross-infection of HCV in dialysis patients who shared dialysis machines in the HD unit.[16,17] Use of dedicated machines along with strict enforcement of universal precautions is associated with a decrease in the incidence of seroconversion.[18] However, a multicenteric study where HCV positive and HCV negative patients were dialyzed on same machines reported no new cases of HCV transmission over a 54 month study period. This study demonstrated complete prevention of HCV transmission by adherence to universal precautions.[19] There are two studies, one each from Belgium and Portugal, which reported comparable incidence of HCV infection in patients treated in units that reprocessed dialyzer.[14,20] In fact a decline in the prevalence of HCV seropositivity among HD patients occurred in the presence of reuse of dialyzer.[21] Similarly, there was no significant impact of dialyzer reuse in our study.

Center for disease control and prevention in the United States (CDC) does not recommend dedicated machines, patient isolation, or a ban on reuse in HD patients with HCV infection.[22] Strict adherence to ‘universal precautions’, careful attention to hygiene, and strict sterilization of dialysis machines have been shown to prevent transmission of infection.[19]

As per literature review, anti-HCV positive HD patients had received significantly more units of blood products than anti-HCV negative patients.[23] The risk of acquiring post-transfusion HCV infection has significantly declined primarily because of availability of better screening test for HCV and erythropoietin.[24] We could not demonstrate any significant association of blood transfusions received and HCV RNA positivity. Our patients come from different economic backgrounds and most of them are self-funded. This leads to compromise in optimum dialysis and EPO dose.

Duration of dialysis has been reported to be significantly longer among anti-HCV positive patients compared to anti-HCV negative patients.[24] Patients on peritoneal dialysis (PD) are at a lower risk for HCV infection. Cendoroglo *et al.*, studied 129 anti-HCV negative patients on chronic dialysis and reported rate of seroconversion of 0.15 per patient-year on HD compared to 0.03 per patient-year on CAPD.[25]

Our results have also emphasized the impact of duration of HD as an important risk factor. Significantly higher risk of HCV RNA positivity is seen after 16 months of HD.

In conclusion, we have high prevalence of HCV RNA in our dialysis population. Duration of HD, getting dialysis at more than one center, elevated ALT, AST, and GGTP, and low serum albumin are found to be important risk factors for HCV RNA positivity. This study is a point prevalence study designed to check actual prevalence of HCV RNA positivity at our center. We accept universal precautions as the most significant measure for control of HCV transmission. However, actual practice of universal precautions by dialysis technicians and nursing staff are probably lacking. We do not isolate HCV positive patients and they are dialyzed on the same machines. Isolation of these patients is difficult because of administrative problems, cross-transmission of various genotypes among the segregated HCV-positive patients, and inability to adopt HCV RNA PCR as screening test due to high cost. Means of transmission of HCV RNA appear to be similar to what other authors have reported. Implementation of universal precautions remains the best way to control transmission of HCV. Dialysis units need to review and rework strategy for controlling transmission of HCV in dialysis population.
